# Safety evaluations of a synthetic antimicrobial peptide administered intravenously in rats and dogs

**DOI:** 10.1038/s41598-022-23841-2

**Published:** 2022-11-11

**Authors:** Laura Cresti, Chiara Falciani, Giovanni Cappello, Jlenia Brunetti, Silvia Vailati, Elsa Melloni, Luisa Bracci, Alessandro Pini

**Affiliations:** 1 Azienda Ospedaliera Universitaria Senese, via M. Bracci, 53100 Siena, Italy; 2 Medical Biotechnology Department, University of Siena, via A Moro 2, 53100 Siena, Italy; 3 SetLance srl, via Fiorentina 1, 53100 Siena, Italy; 4 Zambon spa, via A. Meucci 3, 20091 Bresso, MI Italy

**Keywords:** Diseases, Drug discovery, Drug safety, Pharmacology

## Abstract

The antimicrobial peptide SET-M33 is under study for the development of a new antibiotic against major Gram-negative pathogens. Here we report the toxicological evaluation of SET-M33 administered intravenously to rats and dogs. Dose range finding experiments determined the doses to use in toxicokinetic evaluation, clinical biochemistry analysis, necroscopy and in neurological and respiratory measurements. Clinical laboratory investigations in dogs and rats showed a dose-related increase in creatinine and urea levels, indicating that the kidneys are the target organ. This was also confirmed by necroscopy studies of animal tissues, where signs of degeneration and regeneration were found in kidney when SET-M33 was administered at the highest doses in the two animal species. Neurological toxicity measurements by the Irwin method and respiratory function evaluation in rats did not reveal any toxic effect even at the highest dose. Finally, repeated administration of SET-M33 by short infusion in dogs revealed a no-observed-adverse-effect-level of 0.5 mg/kg/day.

## Introduction

Rising antimicrobial resistance (AMR) is one of the greatest health challenges the world currently faces. It is estimated that approximately 700,000 deaths/year globally are due to drug-resistant bacteria^1,2^. It has been predicted that AMR could cause as many as 10 million deaths/year by 2050 with a global economic burden of USD 100 trillion^3,4^.

Although the need for new antibiotics is urgent, it seems that no single response is sufficient to fight AMR^5,6^. Considering the evolution of resistance to each new class of antibiotics introduced historically and the challenges involved in producing new antibiotics, focusing only on the research and development of new antibiotics is clearly insufficient^7,8^. A concerted global strategy is required.

Antimicrobial peptides (AMPs) are considered an interesting class of antibacterial molecule^9,10^. They cannot be considered a complete alternative to traditional antibiotics because they generally have lower activity and poor stability, and sometimes production difficulties^11–13^. However, they can play a very important role in the fight against bacteria because they are often active against bacteria resistant to traditional antibiotics^14–16^. Furthermore, some have a multifactorial mechanism of action: they kill bacteria and neutralize bacterial toxins, thus greatly reducing the inflammatory process triggered by living and dead bacteria^17,18^.

SET-M33 is a non-natural peptide synthesized in multiple antigen peptide form that makes it more stable in biological fluids^19–21^]. SET-M33 has shown high antimicrobial activity in vitro and in vivo, anti-inflammatory activity, lack of immunogenicity and ability to eradicate biofilms^22,23^. Its mode of action features a two-step mechanism: (1) high affinity binding to LPS^24^ and (2) disruption of bacterial membranes^25^. Data on similar forms of the peptide, such as SET-M33D^26^, SET-M33DIM^27^, SET-M33Peg^28^ and SET-M33 encapsulated in dextran nanoparticles^29^, has been reported with in vivo activity as well. The peptide SET-M33 has completed preclinical development as a new antibacterial agent against major Gram-negative pathogens. Preclinical tests including CMC activities, ADME profile and in vitro and in vivo efficacy in a murine model have been performed.

In this article we report the first toxicity results for SET-M33 administered intravenously in rats and dogs, two animal species recommended as rodent and non-rodent test systems, respectively, by international guidelines^30,31^. We report: dose range finding (DRF) in Sprague Dawley rats and beagle dogs; neurological toxicity and evaluation of respiratory function in rats; a 4-week toxicity study with 2-week recovery period in rats; 4-week toxicity study with 4-week recovery in dogs.

## Results

### Dose range finding (DRF) in rats

The purpose of this study was to determine the DRF of SET-M33 peptide when administered by intravenous bolus to Sprague Dawley rats. In order to identify a starting point for DRF, in Phase I of this study the peptide was administered once daily for 3 days at a constant dose of 20 mg/kg/day to six animals (three males and three females). Detailed observations were made daily during dosing: decreased or increased activity, circling, slow breathing, partially closed eyelids, swollen eyelids, abnormal uncoordinated gait, reduced body tone and hunched posture were observed in treated animals in the first 5 min. No clinical signs were recorded in any animal 20–60 min after administration. Body weight of all animals in Phase I remained constant or decreased slightly during treatment.

Phase II, where 10 animals/group (five males and five females) were used, included a control group and two dose groups (10 mg/kg/day and 20 mg/kg/day), that were injected once daily for seven days. Moderate clinical signs, such as partially closed eyelids, not perfectly coordinate gait and hunched posture, were observed at 10 mg/kg/day. At 20 mg/kg/day, additional clinical signs, including decreased activity, irregular and/or slow breathing, piloerection, partially closed eyelids, abnormal uncoordinated gait, reduced body tone and hunched posture, were observed during the 7 days. The mean body weight of males treated with 10 and 20 mg/kg/day declined slightly (6% and 5% less than control group, respectively). Female body weight was unaffected by treatment. All animals survived both phases.

#### Clinical laboratory investigations

Clinical haematology, biochemistry and urinalysis parameters were evaluated in animals in Phase II at the end of the treatment period. A complete list of parameters is reported in Materials and Methods. Administration of SET-M33 at 10 and 20 mg/kg/day for 7 days caused a statistically significant decrease of reticulocytes in males and females. SET-M33 caused a significant dose-related increase in creatinine levels in both sexes at 10 and 20 mg/kg/day and a significant increase in urea levels in both sexes at 20 mg/kg/day. There were significant differences in other parameters, such as glucose (males at 10 and 20 mg/kg/day), cholesterol (males at 20 mg/kg/day), triglycerides (females at 20 mg/kg/day), electrolytes, total protein and albumin (males at 20 mg/kg/day). Increased urine volume and lower specific gravity compared to the control group was recorded in both sexes at both doses. Differences were statistically significant for specific gravity in all cases and for volume in females at 20 mg/kg/day. Group mean values (± standard deviation) of significant changes are reported in Table 1.

**Table 1:** Group mean values of significant changes obtained in haematology, clinical biochemistry and urinalysis tests (group mean values ± standard deviation) in male and female rats treated intravenously once daily for seven days with 0 (vehicle = 0.9% saline solution), 10 and 20 mg/kg/day of SET-M33 peptide.

Sex	Dose (mg/kg/day)	Haematology		Clinical biochemistry												Urinalysis	
		Retic (× 10^12^/L)	Retic (%)	Urea (mmol/L)	Creat (µmol/L)	Gluc (mmol/L)	Chol (mmol/L)	Trig (mmol/L)	Na (mmol/L)	K (mmol/L)	Cl (mmol/L)	Ca (mmol/L)	Phos (mmol/L)	Total prot (g/L)	Alb (g/L)	SG1	Vol (mL)
Male	0	0.112 ± 0.0088	1.46 ± 0.131	4.82 ± 0.537	24 ± 1.9	12.58 ± 1.155	2.49 ± 0.213	0.81 ± 0.16	134 ± 1.1	4.4 ± 0.26	93 ± 1.2	2.48 ± 2.48	2.51 ± 0.106	55 ± 1.2	30 ± 1.8	1.0299 ± 0.006	7 ± 1.8
	10	0.037** ± 0.007	0.46** ± 0.1	5.46 ± 0.756	41** ± 2.9	8.73** ± 1.741	2.79 ± 0.29	0.73 ± 0.139	136** ± 1.3	4.0* ± 0.18	96** ± 1.3	2.38** ± 0.036	2.31** ± 0.098	57 ± 1.2	31 ± 1	1.0163** ± 0.0044	11 ± 4.5
	20	0.027** ± 0.007	0.33** ± 0.096	8.52** ± 1.286	62** ± 3.3	7.64** ± 0.650	3.46** ± 0.325	0.85 ± 0.183	136** ± 0.4	4.1* ± 0.29	96** ± 0.6	2.54* ± 0.033	2.26** ± 0.081	62** ± 1.3	34** ± 1.6	1.0197** ± 0.0043	13 ± 7.6
Female	0	0.086 ± 0.0109	1.14 ± 0.179	4.77 ± 1.894	27 ± 2.4	8.31 ± 1.051	2.52 ± 0.448	0.57 ± 0.213	137 ± 1.2	3.8 ± 0.24	97 ± 1.5	2.44 ± 0.073	1.92 ± 0.138	57 ± 4	35 ± 1.9	1.0328 ± 0.0173	5 ± 3
	10	0.065 ± 0.0266	0.85 ± 0.323	5.79 ± 0.971	46** ± 6	7.57 ± 0.884	2.99 ± 0.241	0.75 ± 0.127	136 ± 1.1	3.6 ± 0.21	97 ± 1.1	2.46 ± 0.079	1.96 ± 0.187	61 ± 2.8	34 ± 1.5	1.0154* ± 0.0079	18 ± 8.4
	20	0.043** ± 0.008	0.57** ± 0.143	7.63* ± 1.941	57** ± 9.7	7.15 ± 1.529	3.09 ± 0.574	0.93* ± 0.213	136 ± 0.5	3.6 ± 0.36	97 ± 2	2.5 ± 0.54	2.05 ± 0.26	60 ± 3	33 ± 0.9	1.0142* ± 0.0045	20* ± 14.8

*Retic* reticulocyte count (absolute and relative), *Creat* creatinine, *Gluc* glucose, *Chol* total cholesterol, *Trig* triglycerides, *Na* sodium, *K* potassium, *Cl* chloride, *Ca* calcium, *Phos* inorganic phosphorus, *Total Prot* total protein, *Alb* albumin, *SG1* specific gravity, *Vol* volume.

Significant differences between peptide vs. control groups are expressed at 5% (*p < 0.05) or 1% (**p < 0.01) level. For statistical analysis, Dunnett, Shirley or Williams tests were used.

#### Necroscopy

A gross necroscopy examination was performed on Phase I animals. A full necroscopy was performed on all Phase II animals. Organs were collected and weighed. No treatment-related findings were recorded at the end of Phase I. After Phase II, pale kidneys were observed in one female at 10 mg/kg/day and two females at 20 mg/kg/day. Dilated pelvis was observed in one male at 10 mg/kg/day. Higher kidney weight was recorded in both sexes at 20 mg/kg/day (26% and 36% increase with respect to the control group for males and females, respectively). A statistically significant dose-related decrease in heart weight was recorded in treated males compared to the control group. Prostate, seminal vesicles and coagulating gland weight from males dosed at 20 mg/kg/day was significantly lower than in the control group; no differences were recorded in the weight of the testes.

#### Bioanalytic and toxicokinetic study

SET-M33 concentration was determined after administration by intravenous bolus for 7 days at 10 mg/kg/day and 20 mg/kg/day. Exposure parameters (AUC_t_ and C_max_) were compared in order to evaluate dose-dependency, accumulation ratio and sex-related differences. On day 1, SET-M33 profiles showed quantifiable concentrations until 1 h post-dose and on day 7, until 0.5–1 h for 10 mg/kg/day and up to 24 h for 20 mg/kg/day. Mean plasma levels of SET-M33 increased in parallel with dose in males and females. Mean time to maximum concentrations (t_max_) were observed immediately after administration, 5 min post-dose for both periods (days 1 and 7) and sexes, coherently with intravenous bolus administration. On day 1, mean AUC_t_ and C_max_ values for the low vs. high dose were close to the theoretical ratio of 2 (values: 2.2 for AUC_t_ and 2.4 to 2.7 for C_max_). On day 7, mean AUC_t_ and C_max_ values for the low vs. high dose were higher than the theoretical ratio of 2 (values: 9.8–14.0 for AUC_t_ and 2.8–7.2 for C_max_). At 20 mg/kg, exposure to SET-M33 was higher on day 7 than on day 1, whereas no accumulation or low accumulation was observed at 10 mg/kg. Mean SET-M33 exposures were comparable for males and females in all groups and on days 1 and 7 (data not shown).

### Four-week toxicity study with 2-week recovery period in rats

The purpose of this study was to evaluate the toxic effects of SET-M33 when administered intravenously to Sprague Dawley rats for 4 weeks. Recovery was evaluated during a 14-day drug-free period. The peptide was administered once a day at three dose levels at 5, 9 and 15 mg/kg/day, and the control group was treated with the vehicle (ten males and ten females per group which were sacrificed after 4 weeks for clinical and necroscopy analyses; five males and five females for the control and 15 mg/kg/day groups which were sacrificed after the 2 weeks recovery period). The higher dose was selected on the basis of the DRF study (above) in which evident toxicity was recorded at the dose of 20 mg/kg. The efficacy dose in mouse is 5 mg/kg^24^. The lowest dose of this test is approximately twice the equivalent efficacy dose in rat (using body surface area) and the intermediate dose is approximately the geometric mean of the other two.

Mortality was only recorded at 15 mg/kg/day. One male died on day 8 and one male and one female were euthanized for animal welfare reasons on days 6 and 10 of the recovery period, respectively, after showing various clinical signs (hunched back, abnormal gait and pallor) and weight loss of 14% and 11%, respectively. SET-M33-related effects consisting of decreased motor activity, irregular breathing, piloerection, closed or partially closed eyelids, abnormal gait and hunched back were recorded in animals treated at 15 mg/kg/day just after administration for the whole treatment period (as in the DRF study above). From day 8 of recovery onwards, hunched back and pallor were observed in animals treated at 15 mg/kg/day. Among animals that had to be sacrificed for welfare reasons, abnormal gait, partially closed eyelids (the male) and decreased motor activity and piloerection (the female) were also recorded from day 3 onwards. Effects on body weight were observed mainly in males during the treatment period. During the recovery period, body-weight loss (between 2 and 14% based on individual values) was recorded in some animals dosed at 15 mg/kg/day.

#### Clinical laboratory investigations

Blood, coagulation, biochemistry and urinalysis parameters were evaluated at the end of treatment and at the end of recovery. A complete list of parameters can be found in Materials and Methods. A dose-related decrease in red blood parameters (haematocrit, haemoglobin, red blood cells and reticulocyte count) was noted in males and females at all dose levels, but was most pronounced at 15 mg/kg/day. Differences in other parameters, such as mean corpuscular haemoglobin (MCH), mean corpuscular haemoglobin concentration (MCHC), mean corpuscular volume (MCV) and haemoglobin concentration distribution width (HDW) were also observed, mainly at doses of 9 and 15 mg/kg/day. A significant increase in white blood cells (WBC), mainly due to an increase in lymphocyte and large unstained cell (LUC) count was recorded at 15 mg/kg/day, in both sexes. Platelet count was significantly higher than in the control group at all doses in males and females. Differences were dose-related. A slight increase in prothrombin time (SPT) and a decrease in activated partial thromboplastin time (SAPT) were recorded in both sexes at 9 and 15 mg/kg/day (Table 2). The effects on red blood cell parameters were not reversible after 2 weeks of recovery, whereas the effect on lymphocyte count seemed to be reversible, as no significant differences compared to control were recorded in males or females (Table 2). A significant dose-related increase in alkaline phosphatase was recorded at all dose levels in animals treated at 9 and 15 mg/kg/day. Increased gamma-glutamyl-transferase (gGT) was also observed in females at 9 and 15 mg/kg/day. A sharp dose-related effect on urea and creatinine was recorded at all doses, being statistically significant in all cases except in females at 9 mg/kg/day. Urea levels were more than 4 times control group values at 15 mg/kg/day. Creatinine values were more than 6 times control group values at 15 mg/kg/day. A significant decrease in albumin levels (and in total protein levels in males) was observed at 15 mg/kg/day (Table 3). Differences in alkaline phosphatase, gGT (mainly in females), bilirubin, urea and creatinine values had not reversed after 2 weeks of recovery.

**Table 2:** Major changes obtained in haematology and coagulation measurements (group mean values ± standard deviation) in male and female rats treated intravenously for 4 weeks and 2 weeks of recovery period with 0 (vehicle = 0.9% saline solution), 5, 9 and 15 mg/kg/day of SET-M33 peptide.

Sex	Dose (mg/kg/day)	Haematology and coagulation week 4 of treatment—Group mean values														
		Hct (L/L)	Hb (g/dL)	RBC (× 10^12^/L)	Retic (× 10^12^/L)	Retic (%)	MCH (pg)	MCHC (g/dL)	MCV (fL)	WBC (× 10^9^/L)	HDW (g/dL)	L (× 10^9^/L)	LUC (× 10^9^/L)	Plt (× 10^9^/L)	SPT (sec)	**SAPT (sec)**
Male	0	0.454 ± 0.0145	15.3 ± 0.45	8.49 ± 0.32	0.139 ± 0.0233	1.64 ± 0.253	18.00 ± 0.49	33.70 ± 0.71	53.50 ± 0.79	10.00 ± 1.761	2.94 ± 0.097	8.67 ± 1.283	0.08 ± 0.022	779 ± 143.1	23.0 ± 6.54	37.6 ± 5.65
	5	0.401** ± 0.0198	13.5** ± 0.48	7.55** ± 0.306	0.152 ± 0.0283	2.00* ± 0.352	17.90 ± 0.38	33.80 ± 0.76	53.00 ± 1.15	10.76 ± 1.713	3.21** ± 0.126	9.73 ± 1.418	0.08 ± 0.027	917* ± 95.4	21.8 ± 0.82	36.5 ± 6.18
	9	0.348** ± 0.0275	11.9** ± 0.84	6.81** ± 0.538	0.076** ± 0.0292	1.10** ± 0.357	17.40* ± 0.39	34.20 ± 0.73	51.0** ± 1.71	11.36 ± 2.206	3.30** ± 0.174	10.30 ± 2.104	0.10 ± 0.049	923* ± 148.7	24.1** ± 0.68	31.6* ± 5.31
	15	0.311** ± 0.0158	10.7** ± 0.58	6.15** ± 0.322	0.037** ± 0.0091	0.60** ± 0.160	17.50* ± 0.47	34.6** ± 0.50	50.50** ± 0.77	13.51** ± 2.506	3.23** ± 0.151	12.35** ± 2.216	0.14** ± 0.053	961** ± 130.1	24.5** ± 1.05	31.9* ± 5.11
Female	0	0.416 ± 0.0133	14.1 ± 0.47	7.62 ± 0.23	0.176 ± 0.0445	2.31 ± 0.52	18.50 ± 0.40	33.80 ± 0.73	54.70 ± 1.64	7.30 ± 2.064	2.76 ± 0.096	6.25 ± 2.013	0.05 ± 0.023	853 ± 204.900	21.9 ± 1.150	37.0 ± 5.260
	5	0.382** ± 0.0127	12.9** ± 0.46	7.11** ± 0.291	0.180 ± 0.0473	2.55 ± 0.712	18.20 ± 0.40	33.90 ± 0.43	53.70 ± 0.91	7.11 ± 0.596	2.92 ± 0.127	6.31 ± 0.710	0.05 ± 0.011	1004* ± 115.600	21.9 ± 0.660	33.1 ± 5.900
	9	0.352** ± 0.0199	12.0** ± 0.48	6.61** ± 0.358	0.112** ± 0.0462	1.69* ± 0.646	18.10 ± 0.43	34.00 ± 0.80	53.30* ± 0.76	9.15 ± 2.982	3.34** ± 0.189	7.91 ± 2.475	0.08** ± 0.038	1075** ± 136.70	23.6** ± 1.00	26.7** ± 3.890
	15	0.284** ± 0.0223	9.7** ± 0.75	5.44** ± 0.488	0.060** ± 0.0162	1.12** ± 0.345	17.9** ± 0.48	34.20 ± 0.33	52.3** ± 1.44	10.34** ± 1.917	2.99** ± 0.199	9.22** ± 1.713	0.09** ± 0.019	1250** ± 181.00	23.3** ± 1.270	25.6** ± 5.910

Sex	Dose (mg/kg/day)	Haematology and coagulation week 2 of recovery—Group mean values														
		Hct (L/L)	Hb (g/dL)	RBC (× 10^12^/L)	Retic (× 10^12^/L)	Retic (%)	MCH (pg)	MCHC (g/dL)	MCV (fL)	WBC (× 10^9^/L)	HDW (g/dL)	L (× 10^9^/L)	LUC (× 10^9^/L)	Plt (× 10^9^/L)	SPT (sec)	SAPT (sec)
Male	0	0.450 ± 0.0136	15.2 ± 0.4	8.73 ± 0.359	0.122 ± 0.0091	1.40 ± 0.13	17.4 ± 0.61	33.8 ± 0.74	51.6 ± 0.8	9.86 ± 1.818	3.03 ± 0.075	8.39 ± 1.371	0.08 ± 0.026	722 ± 58.2	21.5 ± 0.63	20.3 ± 3.44
	15	0.262** ± 0.0197	9.5** ± 0.55	5.36** ± 0.37	0.061* ± 0.412	1.1 ± 0.797	17.7 ± 0.47	36.1** ± 0.98	49.0 ± 2.37	6.84* ± 1.913	3.25* ± 0.16	6.12 ± 1.655	0.05 ± 0.034	919* ± 169.1	21.1 ± 0.67	13.3* ± 3.41
Female	0	0.431 ± 0.0097	15 ± 0.29	8.04 ± 0.097	0.083 ± 0.0311	1.03 ± 0.385	18.7 ± 0.29	34.9 ± 0.31	53.6 ± 1.07	7.47 ± 1.792	2.64 ± 0.102	6.16 ± 1.375	0.06 ± 0.014	81 ± 135	22.4 ± 0.33	20.4 ± 1.8
	15	0.268** ± 0.0087	9.3** ± 0.34	5.31** ± 0.139	0.063 ± 0.0254	1.17 ± 0.458	17.5** ± 0.62	34.7 ± 0.5	50.6** ± 1.15	7.89 ± 1.712	3.04** ± 0.167	6.58 ± 1.708	0.05 ± 0.018	1208** ± 106.8	22.9 ± 0.79	16.8* ± 1.37

*Hct* haematocrit, *Hb* haemoglobin, *RBC* erythrocyte count, *Retic* reticulocyte count (absolute and relative), *MCH* mean corpuscular haemoglobin, *MCHC* mean corpuscular haemoglobin concentration, *MCV* mean corpuscular volume, *WBC* leukocyte count, total, *HDW* haemoglobin concentration distribution width, *L* lymphocytes, *LUC* large unstained cells, *Plt* platelet (thrombocyte) count, *SPT* prothrombin time, *SAPT* activated partial thromboplastin time.

Significant differences between peptide vs. control groups were expressed at the 5% (*p < 0.05) or 1% (**p < 0.01) level. For statistical analysis, Dunnett, Shirley’ Williams, Wilcoxon and *t* tests were used.

**Table 3:** Major changes in biochemical and urine parameters (group mean values ± standard deviation) in male and female rats treated intravenously for 4 weeks, followed by a 2-week recovery period with 0 (vehicle = 0.9% saline solution), 5, 9 and 15 mg/kg/day of SET-M33 peptide.

Sex	Dose (mg/kg/day)	Biochemistry week 4 of treatment—Group mean values										Urinalysis week 4 of treatment—Group mean values				
		ALP (U/L)	gGT (U/L)	Urea (µmol/L)	Creat (µmol/L)	Na (mmol/L)	Cl (mmol/L)	Ca (mmol/L)	Phos (mmol/L)	Total Prot (g/L)	Alb (g/L)	SG1	Vol (mL)	Prot (g/L)	U-Creat (µmol/L)	U-Gluc (mmol/L)
Male	0	130 ± 19.5	0 ± 0.0	4.91 ± 0.501	23 ± 2.5	138 ± 3.1	98 ± 1.7	2.46 ± 0.095	2.08 ± 0.18	58 ± 3.4	39 ± 2.7	1.051 ± 0.0194	5 ± 2.2	2.51 ± 1.123	12,902 ± 5527.3	4.31 ± 2.798
	5	160** ± 16.8	0 ± 0.2	6.97* ± 0.554	47* ± 4.5	134* ± 2.3	98 ± 1.5	2.34* ± 0.06	1.97 ± 0.309	56 ± 2.4	38 ± 1.8	1.0214** ± 0.0063	10** ± 4	1.04** ± 0.336	4027** ± 1766.3	1.52 ± 0.676
	9	187** ± 28.8	0* ± 0.3	11.64** ± 3.171	97** ± 22.8	134* ± 2.4	103** ± 2.2	2.13** ± 0.088	2.26 ± 0.393	52** ± 3.3	37 ± 4.4	1.0302** ± 0.0219	15** ± 11.3	1.54** ± 1.24	4267** ± 3748.6	11.22 ± 14.93
	15	196** ± 27.8	1** ± 0.3	21.16** ± 7.736	156** ± 59.9	138 ± 3.2	107** ± 3.2	2.11** ± 0.167	2.83** ± 0.324	54** ± 3.5	36* ± 2.4	1.0182** ± 0.003	26** ± 11.9	0.94** ± 0.402	1324** ± 352.0	39.10** ± 13.323
Female	0	98 ± 13.7	0 ± 0.0	5.91 ± 0.963	31 ± 3.2	134 ± 1.9	97 ± 1.6	2.41 ± 0.066	1.84 ± 0.179	56 ± 3.0	41 ± 3.4	1.036 ± 0.0076	6 ± 1.9	0.33 ± 0.186	6205 ± 1731.9	1.68 ± 0.431
	5	111 ± 14.6	0 ± 0.1	6.32 ± 1.258	40 ± 5.8	134 ± 2.2	100* ± 1.6	2.33 ± 0.067	1.45** ± 0.201	58 ± 2.7	42 ± 2.2	1.0203** ± 0.0072	13 ± 4.4	0.30 ± 0.271	2969** ± 1365.2	1.00 ± 0.642
	9	134** ± 12.6	0 ± 0.4	9.81** ± 2.224	80** ± 15	135 ± 1.9	104** ± 2.0	2.36 ± 0.095	1.49 ± 0.415	58 ± 4.7	39 ± 3.2	1.0173** ± 0.0063	20* ± 12	0.34 ± 0.184	1654** ± 667.2	7.77 ± 11.212
	15	119** ± 18.0	2** ± 0.5	28.57** ± 15.12	190** ± 76.6	136 ± 9.8	102** ± 8.6	2.33 ± 0.127	2.52* ± 0.757	55 ± 2.8	36** ± 2.7	1.0175** ± 0.0046	34** ± 13.7	0.76** ± 0.489	985** ± 428.7	43.03** ± 14.984

Sex	Dose (mg/kg/day)	Biochemistry week 2 of recovery—Group mean values										Urinalysis week 2 of recovery—Group mean values				
		ALP U/L	gGT U/L	Urea µmol/L	Creat µmol/L	Na mmol/L	Cl mmol/L	Ca mmol/L	Phos mmol/L	Total Prot g/L	Alb g/L	SG1	Vol mL	Prot g/L	U-Creat µmol/L	U-Gluc mmol/L
Male	0	122 ± 10.9	0 ± 0.0	6.07 ± 0.61	24 ± 1.1	139 ± 0.8	99 ± 0.7	2.6 ± 0.043	1.96 ± 0.122	62 ± 1.6	40 ± 1.4	1.0394 ± 0.0302	7 ± 3.2	1.65 ± 1.52	11,288 ± 8063.3	3.38 ± 4.649
	15	208** ± 53.1	0 ± 0.8	46.64* ± 30.424	235* ± 101.4	145* ± 5.9	104 ± 5.7	2.19** ± 0.154	2.83 ± 1.37	61 ± 4.4	42 ± 3.3	1.0131* ± 0.0004	34** ± 7.5	0.35* ± 0.132	1339* ± 134.3	27.05** ± 5.353
Female	0	105 ± 22.1	0 ± 0.0	5.73 ± 0.558	30 ± 2.0	140 ± 0.7	101 ± 1.3	2.64 ± 0.048	1.65 ± 0.267	64 ± 1.4	44 ± 2.7	1.0508 ± 0.0238	5 ± 2.8	0.78 ± 0.516	11,012 ± 4985.4	2.9 ± 1.896
	15	146* ± 30.0	1* ± 0.8	34.83** ± 16.358	179** ± 63.2	143 ± 4.3	103* ± 1.4	2.45** ± 0.054	2.04 ± 0.616	63 ± 2.0	43 ± 1.4	1.0130* ± 0.0005	34** ± 4.7	0.29 ± 0.206	1089* ± 71.3	34.76** ± 6.659

*ALP* alkaline phosphatase, *gGT* gamma-glutamyl-transferase, *Creat* Creatinine, *Na* sodium, *Cl* chloride, *Ca* calcium, *Phos* inorganic phosphorus, *Total Prot* total protein, *Alb* albumin, *SG1* specific gravity, *Vol* volume, *Prot* protein, *U-Creat* creatinine, *U-Gluc* glucose.

Significant differences between peptide vs. control groups were expressed at the 5% (*p < 0.05) or 1% (**p < 0.01) level. For statistical analysis the Dunnett, Shirley, Williams, Wilcoxon and *t* tests were used.

A higher dose-related volume of urine than in the control group was observed at all doses. The specific gravity recorded in all SET-M33-treated groups was lower than in controls. Differences were statistically significant except for volume in females at 5 mg/kg/day. Creatinine levels lower than in controls were recorded at all doses. Differences were dose-related in females. The protein levels recorded in males from all SET-M33-treated groups were significantly lower than in controls. Statistically significant differences in glucose increase were found in animals treated at 15 mg/kg/day. Differences observed at the end of treatment persisted at the end of the recovery period. Group mean values of significant changes are reported in Table 2 and Table 3.

#### Necroscopy

Necroscopy and histopathological examinations were performed at the end of treatment and at the end of the recovery period. Macroscopic changes were recorded in the kidneys (pale coloration and a reddened medullary area in almost all males and females from groups on 9 and 15 mg/kg/day) and bladder (distended in a few males of all treated groups). These findings were also recorded at the end of the recovery period affecting all animals dose at 15 mg/kg/day (pale coloration of the kidneys) or one male (distended urinary bladder). The mean weight of the kidneys, liver and spleen was significantly higher with respect to control group in both sexes at all dose levels. At the end of the recovery period the mean weight of the spleen (both sexes) and the mean weight of the kidneys and ovaries (females) from animals treated at 15 mg/kg/day were still higher with respect to control group. The microscopic examination revealed SET-M33-related findings in kidneys (all dose levels), femorotibial growth plate, brain, duodenum and ovaries (doses of 15 and 9 mg/kg), and in bone marrow, heart, uterine cervix and vagina and mammary glands (dose of 15 mg/kg). For 15 mg/kg/day group, the changes observed in the kidneys, bone marrow, uterine cervix and vagina were not reversible (see Supplementary Material for images and number of animals affected by these irreversible abnormalities), while the rest of the findings were fully (femorotibial growth plate and duodenum) or partially recovered (heart, ovaries and mammary glands) after 2 weeks of recovery period.

### Neurological toxicity in rats

The purpose of this study was to evaluate the neurological side-effects of SET-M33 following a single intravenous injection of 5, 9 and 15 mg/kg and vehicle (6 animals/group). The study was based on subjective observation of central nervous system effects on general behaviour (Irwin test), combined with objective evaluation of body temperature and locomotor activity in male rats^32,33^. Effects on body weight were also assessed. Again the dose levels were selected on the basis of the DRF study reported above.

SET-M33 did not cause any adverse clinical signs during detailed observations at 5, 30, 90, 240 min and 24 h post-dose. Animals were also inspected daily from day 3 to day 7 for any delayed effects. The list of parameters and observations considered in the Irwin test can be found in Materials and Methods. No delayed effects of toxicity and no mortality were recorded up to day 7.

Pre-dose group mean body temperatures were similar across groups. A single intravenous injection of SET-M33 at doses of 5, 9 and 15 mg/kg had no significant effects on body temperature when compared with control animals. Actually, significantly lower body temperatures were recorded 5 min post-dose in the 9 and 15 mg/kg SET-M33-treated groups compared with control animals (*p < 0.05, **p < 0.01). However, since the group mean values were only 0.4–0.6 °C lower than the vehicle-treated control group, this cannot be considered biologically noteworthy (Table 4).

**Table 4:** Effects of intravenous bolus injection of SET-M33 at 0 (vehicle = 0.9% saline solution), 5, 9 and 15 mg/kg on body temperature, locomotor activity and body weight in male rats (group mean values ± standard deviation).

Dose (mg/kg)	Group mean body temperature (°C ± sd) at time post-dose						Group mean locomotor activity (^#^ ± sd) at time post-dose:						Group mean bodyweight (g ± sd) on day			
	Pre-dose	5 min	30 min	90 min	240 min	24 h	Pre-dose	5 min	30 min	90 min	240 min	24 h	1	2	3	7
0	37.3	38	37.4	37.1	36.6	36.9	44.8	39.3	28.8	27.8	16	35	204.5	206	210.7	229
	± 0.26	± 0.36	± 0.25	± 0.45	± 0.41	± 0.38	± 6.77	± 10.46	± 10.38	± 5.64	± 7.29	± 8.65	± 6.72	± 8.79	± 9.07	± 12.03
5	37.5	37.9	37.2	36.9	36.9	37.1	37.8	30.7	26.5	24.2	22.5	30	207.2	209	215.8	236.5
	± 0.31	± 0.27	± 0.51	± 0.29	± 0.48	± 0.51	± 9.56	± 6.56	± 7.12	± 6.71	± 5.05	± 15.02	± 2.79	± 3.22	± 3.87	± 8.17
9	37.5	37.6*	37.3	36.9	36.7	36.8	38.2	32.5	27	21.3	22.5	28.3	204.8	201.2	209.5	229.5
	± 0.34	± 0.31	± 0.43	± 0.35	± 0.41	± 0.79	± 7.73	± 5.96	± 9.34	± 12.65	± 12.82	± 12.82	± 4.92	± 4.36	± 3.39	± 5.01
15	37.2	37.4**	36.9	36.9	36.7	36.9	38.3	22.2**	29	20.7	18.5	25.5	206	200.3	209.7	227
	± 0.51	± 0.29	± 0.41	± 0.24	± 0.31	± 0.5	± 4.08	± 9.5	± 5.8	± 8.02	± 8.17	± 10.21	± 5.93	± 6.92	± 7.58	± 8.34

Statistical significance compared with vehicle: *p < 0.05, **p < 0.01. The results of each treated group were compared with those of the vehicle-treated control group by analysis of variance and Williams test.

^#^Number of times the rats entered a new square in the arena.

Pre-dose group mean locomotor activity was relatively similar across all groups. SET-M33 at doses of 5 and 9 mg/kg did not cause any effect on locomotor activity when compared with control animals. The 15 mg/kg dose caused a small transient reduction in locomotor activity at 5 min post-dose. However, since this effect subsided by 30 min and only occurred at a single time point, it was not considered to be adverse or dose-limiting (Table 4).

No statistically significant effects on body weight were recorded at any dose. Small weight losses (approximately 2–3%) were recorded in the 9 and 15 mg/kg treated groups at 24 h, compared with the weights of the previous day (not significant with respect to the control group), and were followed by increases on day 3. As these effects were very transient and small in magnitude, they were not considered adverse (Table 4).

No other adverse effects were observed for any other neurological parameter listed in Material and Methods.

### Evaluation of respiratory function in rats

The possible side-effects of SET-M33 on respiratory function were evaluated after administration of the peptide as a single intravenous bolus at doses of 5, 9 and 15 mg/kg with vehicle (eight animals/group). Respiratory rate, tidal volume and minute volume were assessed. As in the case of neurological toxicity and the 4-week toxicity study, again the dose levels were selected on the basis DRF (above). Baclofen, a muscle relaxer and antispasmodic used to treat muscle pain, spasms and stiffness in people with multiple sclerosis or spinal cord injury or disease, was used as positive control. It was administered as a single intravenous injection at a dose of 15 mg/kg.

**Figure 1:**
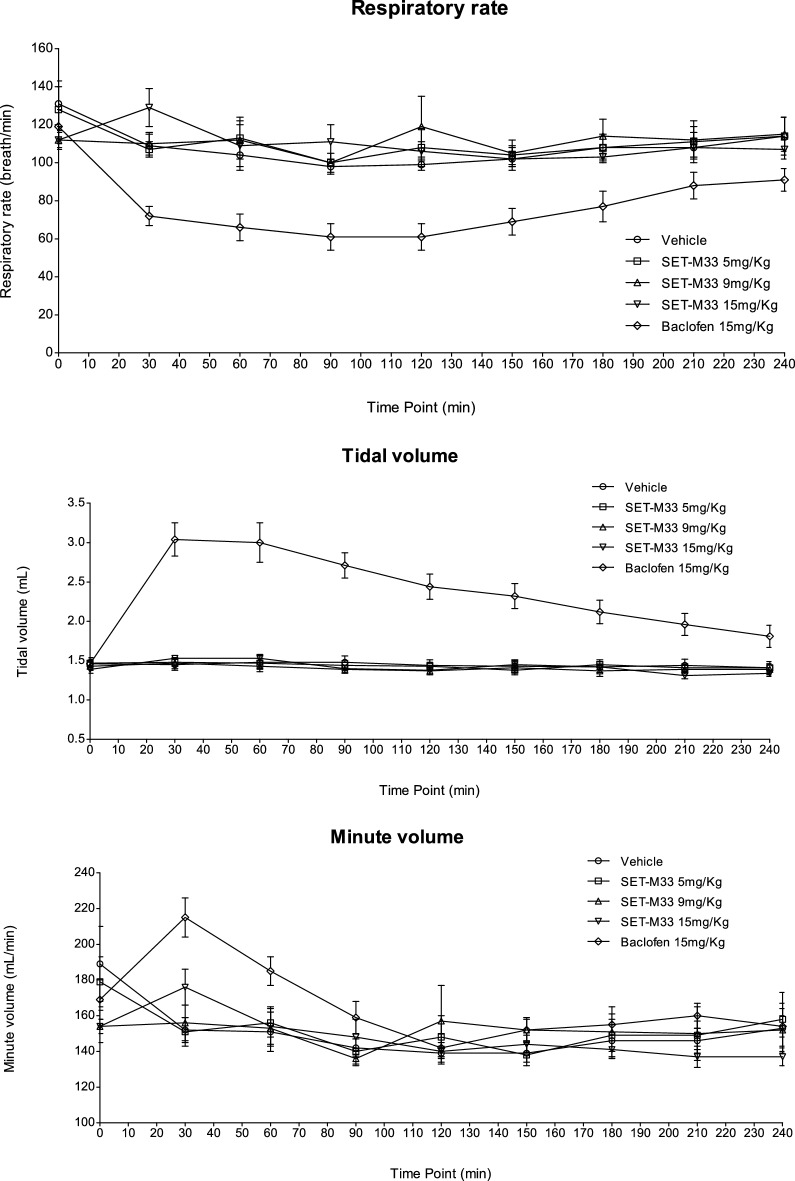
Effects of intravenous injection of SET-M33 at 0 (vehicle), 5, 9 and 15 mg/kg on respiratory rate (breaths/min ± se), tidal volume (mL ± se) and minute volume (mL/min ± se) in rats. Baclofen at 15 mg/day was used as positive control. *se* standard error of mean. Symbols (triangle, circle, rhombus or square) indicate a group mean respiratory parameter. The bar represents the standard error. The graphs were obtained using GraphPad Prism for Windows version 5.03, GraphPad Software, San Diego, California USA, www.graphpad.com. The groups treated with SET-M33 peptide were compared to vehicle using Williams’ test. The comparison between positive control and vehicle were made using two-tailed *t* test based on the error mean square from the analysis of covariance.

SET-M33 did not produce any statistically significant adverse effects on respiratory rate, tidal volume or minute volume with respect to control animals, except for a statistically significant increase in minute volume recorded at 30 min post-dose at 15 mg/kg. However, since this effect subsided by 60 min post-dose and only occurred at a single time point, it was not considered to be adverse or dose-limiting. Baclofen produced sharp, statistically significant increases in tidal volume and minute volume, which were accompanied by a sharp, statistically significant decrease in respiratory rate, compared with vehicle-treated control animals (Fig. 1). At 30 min the group treated at 15 mg/kg had significantly higher minute volume than the vehicle control (*p* = 0.019, Williams’ test). At 30 and 60 min the group treated with the Baclofen had significantly higher minute volume than the vehicle control (*p* ≤ 0.014, *t* test). At all post-dose time points the group treated with Baclofen had significantly higher tidal volume than the vehicle control (*p* < 0.001, *t *test). At 30, 60, 90, 120, 150, 180 and 240 min the group treated with Baclofen had significantly lower respiration rate than the vehicle control (*p* ≤ 0.031, *t* test).

### Dose range finding (DRF) in dogs

The purpose of this study was to conduct DRF for SET-M33 administered by short intravenous infusion (30 min) to beagle dogs at 4.0 and 8.0 mg/kg/day (7-day treatment period). Two animals served as control group (one male, one female), and four animals were assigned to treated groups (two males, two females). The doses were determined by a preliminary study not reported here.

The following clinical signs were reported: tremors were occasionally recorded in a male treated at 4 mg/kg (on days 4 and 5 of administration), and in a male and a female treated at 8 mg/kg (on days 5 of administration). In the males treated at 8 mg/kg, vomit was observed in the box on days 1 (both animals) and 3 (one dog). A 9.4% loss of body weight with respect to day 1 was recorded at the end of treatment in one animal of the 8 mg/kg/day group. No noteworthy effects on body weight were observed in the other dogs. All animals survived the scheduled period.

#### Clinical laboratory investigations

Blood, coagulation, biochemical and urine parameters were evaluated pre-test and on completion of treatment. The complete list of parameters evaluated is reported in Materials and Methods. Increased urea and creatinine levels were recorded in males and females on 8 mg/kg/day (67% and 74% higher than pre-test values for males, and 26% and 21% higher than pre-test values for females, respectively). No noteworthy effects on blood and coagulation parameters were recorded. Group mean values of significant changes are reported in Table 5.

**Table 5:** Major differences of urea and creatinine in clinical biochemistry values (group mean values ± standard deviation) in beagle dogs treated with intravenous 30 min-short infusion of SET-M33 at 0 (vehicle = 0.9% saline solution), 4.0 and 8.0 mg/kg/day (7-day treatment period).

Sex	Dose (mg/kg/day)	Urea (nmol/L)		Creatinine (µmol/L)	
		Pre-dose	End of treatment	Pre-dose	End of treatment
Male	0	5.15	4.58	79	58
	4.0	4.31 ± 0.849	4.92 ± 0.035	84 ± 18.4	77 ± 2.8
	8.0	4.26 ± 0.064	7.64 ± 0.191	66 ± 4.2	115 ± 25.5
Female	0	4.57	5.2	58	58
	4.0	3.76 ± 0.156	4.76 ± 0.021	69 ± 17.7	79 ± 2.8
	8.0	6.29 ± 2.15	7.94 ± 0.099	85 ± 23.3	103 ± 0

Statistical analysis was not performed due to the small number of animals.

#### Necroscopy

A full necropsy was performed in all animals. Organs were collected and weighed. No noteworthy changes in organ weight were reported for the doses tested, except for lower weight of the thymus in males on doses of 4 and 8 mg/kg/day (66 and 76% less than control, respectively).

#### Bioanalytic and toxicokinetic study

Concentrations of SET-M33 in dog plasma samples and the main toxicokinetic parameters were determined. The exposure parameters (AUC_t_ and C_max_) were compared to evaluate dose-dependency, accumulation ratio and sex-related differences. Overall, on day 1, SET-M33 profiles observed at doses of 4 and 8 mg/kg/day showed quantifiable concentrations until 3.5 h after starting the infusion. In addition, one male on 8 mg/kg/day showed quantifiable concentrations at day 7 up to 24.5 h after the start of infusion. Mean SET-M33 plasma levels increased in parallel in males and females. Mean time to maximum SET-M33 concentration (t_max_) was after the end of infusion for both periods (days 1 and 7) and sexes, i.e. 30 min post-dose, coherently with intravenous administration. On day 1 and 7, mean AUC_t_ and C_max_ values for the low vs. high dose were close to the theoretical ratio of 2. No accumulation or low accumulation was observed at 4 and 8 mg/kg after 7 days. Mean SET-M33 exposures were comparable for males and females in all groups on days 1 and 7, with a male/female ratios ranging from 0.3 to 1.3 for C_max_ and from 0.4 to 1.3 for AUC_t_ (data not shown).

### Four-week toxicity study with 4-week recovery period in dogs

Beagle dogs (5/sex/group) were dosed by intravenous infusion once daily for 1 h with 0 (0.9% sodium chloride for injection), 0.5, 1.5 or 4.0 mg/kg/day SET-M33 for four consecutive weeks. The doses were selected in the dog DRF study (above). The dose of 4 mg/kg/day was selected as the highest dose without macroscopic side effects in the DRF study, and was used to evaluate renal toxicity and its reversibility. The dose of 0.5 mg/kg/day was selected as the lowest dose, about one order of magnitude less than the highest dose. The dose of 1.5 mg/kg/day was selected as an approximately median mid-dose. On completion of 4 weeks of infusion, three animals/sex/group were euthanized and necropsied and the remaining two animals/sex/group were held for a 4-week drug free recovery period after which they were euthanized and necropsied. Blood samples were obtained from all animals on days 1 and 28 for toxicokinetic analysis.

All animals survived until their scheduled termination and there were no noteworthy peptide-related clinical signs.

#### Clinical laboratory investigations

Blood, coagulation, biochemical and urine parameters were evaluated. A complete list of parameters is reported in Materials and Methods. There were no SET-M33-related blood or coagulation changes at the end of treatment or in the 4-week recovery period. SET-M33-related biochemical changes at all doses in both sexes included increases in blood urea nitrogen (BUN; 25–64% higher than pre-test) and creatinine (14–50% higher than pre-test) in individual animals (data not show). The magnitude of these changes was generally similar across all dose groups. These increases were correlated with minimal renal tubule degeneration/regeneration at 1.5 mg/kg/day and 4 mg/kg/day in males and at 4.0 mg/kg/day in females and minimal interstitial cell infiltrate in both sexes at a dose of 4.0 mg/kg/day. There were no SET-M33-related biochemical changes at any dose after 4-week recovery, indicating complete recovery of the changes detected at the end of dosing. There were no SET-M33-related urine changes at 0.5 or 1.5 mg/kg/day. There were decreases in urine specific gravity and urine creatinine concentrations with respect to individual pre-test values in individual animals on 4.0 mg/kg/day. These changes were more prominent in females and suggest more dilute urine, possibly associated with a lower concentrating ability of the kidneys. There were no SET-M33-related urine changes at any dose after the 4-week recovery period.

#### Necroscopy

Higher SET-M33-related kidney weights (absolute and relative to body and brain weight) were observed in both sexes at a dose of 4.0 mg/kg/day. These higher weights were correlated microscopically with minimal tubule degeneration/regeneration and minimal interstitial cell infiltrates. There were no other SET-M33-related organ weight changes (Table 6). There were no organ weight changes at recovery sacrifice, or macroscopic findings at terminal and recovery necroscopy. SET-M33-related microscopic findings were observed in kidneys at doses of 1.5 mg/kg/day and 4 mg/kg/day and at infusion sites at all doses. The kidney findings included minimal tubule degeneration/regeneration at 1.5 mg/kg/day and 4 mg/kg/day in males, and at 4.0 mg/kg/day in females, and minimal interstitial cell infiltrate in both sexes at a dose of 4.0 mg/kg/day. Degeneration featured vacuolation, cell sloughing and/or tinctorial change, and regeneration was characterized by increased basophilia, nuclear crowding and/or increased mitoses of cortical tubule epithelial cells. Regenerative changes were typically more pronounced than degenerative changes. SET-M33-related microscopic findings in kidney were correlated with higher kidney weights (absolute and relative to body and brain weight) and elevated BUN and creatinine in both sexes at 4.0 mg/kg/day. SET-M33-related findings were observed at all four infusion sites (different site each week) in both sexes at all doses and included vascular thrombi (minimal to high), vascular/perivascular inflammation (minimal to moderate), hypertrophy/hyperplasia of the tunica intima or tunica media (minimal to slight), and/or vascular/perivascular haemorrhage (minimal to moderate). The other SET-M33-related microscopic findings in kidney and at infusion sites were considered non-adverse due to the magnitude of the changes (minimal to moderate) and/or a lack of correlations suggesting functional impairment. After the 4-week recovery period, kidney interstitial infiltrates cleared completely and tubule degeneration/regeneration resolved almost completely. At the infusion sites, there was complete recovery from vascular/perivascular inflammation and tunica intima hypertrophy/hyperplasia with partial recovery from vascular thrombi and hypertrophy/hyperplasia of the tunica media. Minimal regeneration of cortical tubule epithelial cells, observed in the kidneys of 1 female at a dose of 4.0 mg/kg/day, was considered to be due to a repair process, and a slight thrombus observed at the infusion site (saphenous vein) of another female was also in line with ongoing repair. In addition, slight tunica media hypertrophy/hyperplasia of the left cephalic vein was observed in one male (Table 6). Slight tunica media hypertrophy/hyperplasia of the left cephalic vein was observed in 1 male at a dose of 4.0 mg/kg/day.

**Table 6:** SET-M33-related changes in beagle dogs dosed with the peptide at 0 (vehicle = 0.9% saline solution), 0.5, 1.5 and 4.0 mg/kg/day for 28 days and after 4-week recovery.

			Male				Female			
			0 mg/kg/day	0.5 mg/kg/day	1.5 mg/kg/day	4.0 mg/kg/day	0 mg/kg/day	0.5 mg/kg/day	1.5 mg/kg/day	4.0 mg/kg/day
**Kidney weight changes (% difference relative to controls)**										
Kidney	Absolute weight (%)			–	–	8.63		–	–	15.16
	vs. body weight (%)			–	–	11.47		–	–	9.56
	vs. brain weight (%)			–	–	9.80		–	–	13.59
**SET-M33-related findings in the kidney after 28-days dosing and after the 4-week recovery period**										
4-week dosing	Degeneration/regeneration, tubules	Minimal	0	2	3	0	0	0	0	2
	Infiltrate, interstitium	Minimal	0	0	1	0	0	0	0	1
4-week recovery period	Regeneration, tubules	Minimal	0	0	0	0	0	0	0	1
**SET-M33-related findings at infusion site after 28-days dosing and after the 4-week recovery period (combined incidence from all four sites)**										
4-week dosing	Thrombus, blood vessels	Minimal	0	1	0	0	0	0	0	0
		Slight	0	0	0	2	0	1	0	0
		Moderate	0	0	0	5	0	0	1	5
		High	0	0	0	1	0	0	0	0
		Total	0	1	0	8	0	1	1	5
	Inflammation, vascular/perivascular	Minimal	0	2	3	0	1	0	1	1
		Slight	0	1	2	0	0	0	1	4
		Moderate	0	0	0	0	0	0	0	1
		Total	0	3	5	0	1	0	2	6
	Hypertrophy/hyperplasia, tunica intima	Minimal	0	0	0	0	1	0	0	0
		Slight	0	1	1	0	0	0	0	0
		Total	0	1	1	0	1	0	0	0
	Hypertrophy/hyperplasia, tunica media	Minimal	0	0	0	1	0	0	0	0
		Slight	0	2	0	2	0	0	1	0
		Total	0	2	0	3	0	0	1	0
	Haemorrhage, vascular/perivascular	Minimal	0	1	1	0	2	1	3	2
		Slight	0	1	0	1	2	1	0	3
		Moderate	0	0	0	0	0	0	1	0
		Total	0	2	1	1	4	2	4	5
4-week recovery period	Thrombus, blood vessels	Slight	0	0	0	0	0	0	0	1
		Total	0	0	0	0	0	0	0	1
	Hypertrophy/hyperplasia, tunica media	Slight	0	0	0	1	0	0	0	0
		Total	0	0	0	1	0	0	0	0

For kidney weight changes statistical analysis was not performed due to the small number of animals.

As a final result of this toxicological study, the no-observed-adverse-effect-level (NOAEL) of SET-M33 was determined to be 0.5 mg/kg/day.

### Bioanalytics and toxicokinetics in rats and dogs

Unlike similar tests reported in DRF studies, here SET-M33 was administered for a prolonged period (4 weeks instead of 1 week) and at doses previously selected by DRF. In order to better relate the toxicokinetics data (presented below) with SET-M33 activity we report, as examples, the following SET-M33 MIC values: MIC_50_ and MIC_90_ for *P. aeruginosa* are 1.4 µM; MIC_50_ and MIC_90_ for *K. pneumoniae* are 1.4 µM and 2.8 µM, respectively^24^.

#### RATS

**Figure 2:**
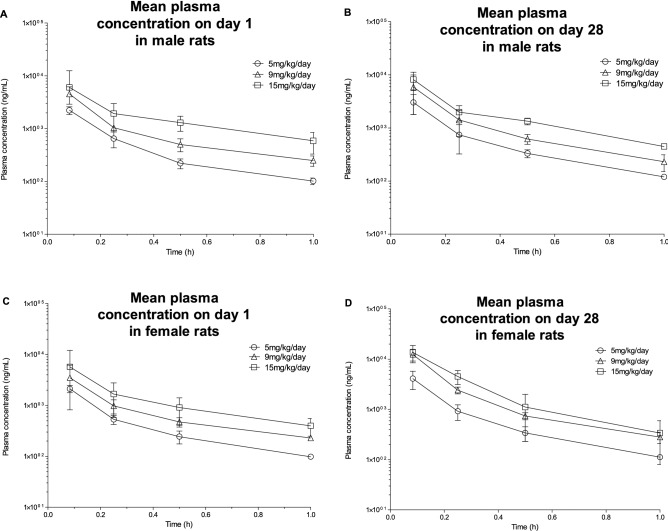
Mean plasma concentrations of SET-M33 on day 1 (**A**) and (**C**) and day 28 (**B**) and (**D**) of 4 weeks of daily intravenous (slow bolus) administration to male (**A**) and (**B**) and female (**C**) and (**D**) rats at 5, 9 and 15 mg/kg/day. Symbols (circle, triangle or square) indicate a group mean value. Bars represent the standard deviation. The graphs were plotted using GraphPad Prism for Windows version 5.03, GraphPad Software, San Diego, California USA, www.graphpad.com.

SET-M33 was administered intravenously as a slow bolus to Sprague Dawley rats once daily for 4 weeks at 0 (vehicle only), 5, 9 and 15 mg/kg/day (control group 3 animals/sex, treated groups 6 animals/sex). Blood samples were taken on day 1 and day 28. The SET-M33 profiles showed quantifiable concentrations until 1 h post-dose at days 1 and 28. Mean plasma SET-M33 exposures were higher for females than males at day 28, while no differences were observed at day 1. Mean plasma SET-M33 exposure increased in parallel with dose in males and females. Males and (especially) females showed higher exposure levels at day 28 than day 1 (Fig. 2). Mean time to maximum plasma SET-M33 concentration (t_max_) was 5 min post-dose for both times and sexes as expected for intravenous bolus administration. On day 1, mean AUC_0-t_ and C_max_ values for the low vs. intermediate and high doses were close to the theoretical ratios of 1.8 and 3 (2 and 2.9–3.2 for AUC_0-t_, and 1.7–2 and 2.7 for C_max_). On day 28, female intermediate dose AUC_0-t_ ratio was 1.7 times the theoretical ratio. The results suggest accumulation of SET-M33 in all dose groups in males and especially in females. This accumulation does not seem due to the repeated dose, since no steady state was reached (Table 7).

**Table 7:** Pharmacokinetic parameters of SET-M33 on day 1 and day 28 of 4 weeks of daily intravenous (slow bolus) administration to male and female rats at 5, 9 and 15 mg/kg/day (group mean values).

Time	Parameters	Units	5 mg/kg/day		9 mg/kg/day		15 mg/kg/day	
			Male	Female	Male	Female	Male	Female
Day 1	t_max_	minutes	5	5	5	5	5	5
	C_max_	ng/mL	2227	2107	4563	3503	6043	5727
	AUC_0-t_	ng ∙ h/mL	692	663	1433	1152	2236	1944
Day 28	t_max_	minutes	5	5	5	5	5	5
	C_max_	ng/mL	3017	4103	5890	12,387	8170	13,567
	AUC_0-t_	ng ∙ h/mL	938	1218	1819	3558	2734	4111

*C*_max_ maximum plasma concentrations, *t*_max_ time at which C_max_ occurred, *AUC*_0-t_ area under the curve from zero to last quantifiable sampling time. Statistical analysis is not applicable to these experiments.

#### Dogs

Plasma concentrations and the main toxicokinetic parameters of SET-M33 in plasma samples were determined in beagle dogs after intravenous administration of SET-M33 by daily 1-h-infusion at 0.5, 1.5 or 4.0 mg/kg/day or only vehicle for 4 weeks (5 animals/group/sex). Blood samples were taken on day 1 and day 28.

**Figure 3:**
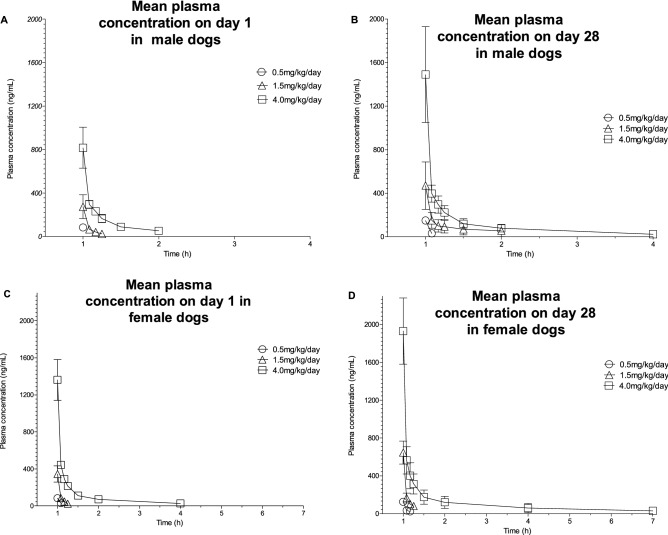
Mean plasma concentrations of SET-M33 on day 1 (**A**) and (**C**) and day 28 (**B**) and (**D**) of 4 weeks of daily intravenous (infusion) administration to male (**A**) and (**B**) and female (**C**) and (**D**) dogs at 0.5, 1.5 and 4.0 mg/kg/day. Symbols (circle, triangle or square) indicate a group mean value. Bars represent standard deviations. The graphs were plotted using GraphPad Prism for Windows version 5.03, GraphPad Software, San Diego, California USA, www.graphpad.com.

Mean plasma concentration–time profiles showed higher values for all doses at day 28 (Fig. 3). Maximum plasma concentrations (C_max_) of SET-M33, their times of occurrence (T_max_) and the areas under the plasma SET-M33 concentration–time curves within a 24-h dosing interval (AUC_0-24_) on day 1 and day 28 are shown in Table 8, where the mean C_max_ and AUC_0-24_ for the group are shown with standard deviations in brackets. The time when the maximum plasma concentration occurred (T_max_) was at the end of the 1-h infusion in all animals, as expected for this route of administration. Plasma concentrations of SET-M33 at 24 h post-dose were below the limit of quantification (< 20.0 ng/ml) in all animals at all dose levels on day 1 and day 28 (not shown).

**Table 8:** Pharmacokinetic parameters of SET-M33 on day 1 and day 28 of 4 weeks of daily intravenous (infusion) administration to male and female beagle dogs at 0.5, 1.5 and 4.0 mg/kg/day (group mean values ± standard deviation of C_max_ and AUC_0-24_).

Time	Parameters	Units	0.5 mg/kg/day		1.5 mg/kg/day		4 mg/kg/day	
			Male	Female	Male	Female	Male	Female
Day 1	t_max_	minutes	EOI	EOI	EOI	EOI	EOI	EOI
	t_last_	minutes	EOI	5	15	15	60	180
	C_max_	ng/mL	82.4 ± 22.4	82.8 ± 19.1	274 ± 110	345 ± 87	816 ± 188	1360 ± 220
	AUC_0-24_	ng ∙ h/mL	42.2 ± 13.3	45.6 ± 10.4	157 ± 60	196 ± 48	569 ± 99	991 ± 161
Day 28	t_max_	minutes	EOI	EOI	EOI	EOI	EOI	EOI
	t_last_	minutes	10	10	60	60	180	360
	C_max_	ng/mL	149 ± 16	125 ± 35	470 ± 218	645 ± 121	1490 ± 440	1930 ± 350
	AUC_0-24_	ng ∙ h/mL	83.0 ± 9.8	76.5 ± 25.2	416 ± 209	402 ± 104	1050 ± 150	1550 ± 450

*AUC*_0-24_ area under the plasma concentration–time curve in 24-h dosing intervals, *C*_max_ maximum plasma concentrations, *EOI* end of infusion, *t*_last_ time point of the last quantifiable plasma concentration, *t*_max_ time at which C_max_ occurred. Statistical analysis is not applicable to these experiments.

**Figure 4:**
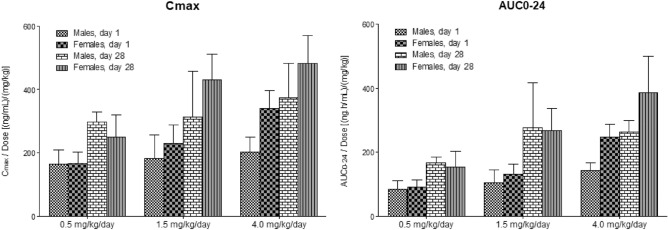
Mean maximum plasma concentrations (C_max_) of SET-M33 and mean areas under the plasma concentration–time curves (AUC_0-24_), on day 1 and day 28 of 4 weeks of daily intravenous (infusion) administration of SET-M33 to beagle dogs at 0.5, 1.5 and 4.0 mg/kg/day. *AUC*_0-24_ area under the plasma concentration–time curve in 24-h dosing intervals, *C*_max_ maximum plasma concentrations. Columns and bars represent group mean values and standard deviations, respectively. The graphs were plotted using GraphPad Prism for Windows version 5.03, GraphPad Software, San Diego, California USA, www.graphpad.com. Statistical analysis is not applicable to these experiments.

The systemic exposure (C_max_ and AUC_0-24_) of dogs to SET-M33 increased with increasing dose over the dose range 0.5–4.0 mg/kg/day on day 1 and day 28. Excluding C_max_ values in males, the C_max_ and AUC_0-24_ values at the highest dose (4.0 mg/kg/day) were approximately 2.1 times higher than those predicted in the case of a linear relationship (not shown). The C_max_ and AUC_0-24_ values of SET-M33 in female dogs were similar to the indices of exposure in males at the two lower dose levels, but were approximately 1.6 times higher than those of males at the highest dose level (Fig. 4). After repeated doses (day 28), C_max_ and AUC_0-24_ values of SET-M33 were generally higher than those after a single dose (day 1) (Fig. 4). The accumulation ratios, based on C_max_ and AUC_0-24_ values, were generally greater than one, indicating that systemic exposure to SET-M33 was higher after repeated administrations than after a single dose (not shown). However, since plasma concentrations of SET-M33 were below the limit of quantification 24 h post-dose in all animals, these results indicate that SET-M33 has time-dependent kinetics.

## Discussion

The standard procedures indicated by regulatory agencies specify that before a new therapeutic entity can be given to humans, developers must first test it thoroughly in animals for safety and efficacy^34^. The main aims of pre-clinical studies are roughly to determine: (i) the efficacy and toxicity of the compound; (ii) its pharmacokinetics; (iii) the formulation for appropriate delivery in humans. Pre-clinical toxicological studies must be conducted in two animal species, including a rodent and a non-rodent, before proceeding to the clinical phases of development^35,36^.

In the preclinical phase, the ultimate goal is to translate the animal model responses into an understanding of the risk for human subjects^37^. Toxicity testing in animals is therefore valuable for lead compound characterization and further decisions about the direction of development^38^.

The peptide SET-M33 is a synthetic molecule under study for the development of a new antibacterial drug. SET-M33 and some of its back-up molecules have already been reported for antibacterial efficacy in different infections and inflammation models in vivo and ex vivo, including sepsis^22,24,26^, pneumonia^24,27,29,39^ and skin infections^24^. As a novel drug to administer intravenously, SET-M33 has entered a preclinical development phase that includes scale up of production, adsorption, excretion^24^ and finally toxicity, the subject of the present report. In the present study, SET-M33 showed a grade of toxicity, which when combined with the efficacy experiments already reported, suggests that it will have a favourable therapeutic index.

When tested in rats, even at highest dose used, SET-M33 did not have any neurological effects as assessed by Irwin tests of behavioural and physiological states, nor did it affect body temperature, locomotor activity or body weight. Likewise, SET-M33 did not affect lung function in terms of respiratory rate, tidal volume and minute volume when compared with the vehicle-treated control and a baclofen-treated positive control.

In prolonged treatment (4-week daily administration and 2-week recovery period) of rats, clinical signs during the recovery period and loss of body weight recorded in some animals showed that animals treated with the high dose (15 mg/kg/day) were unable to recover. Mean plasma concentrations quantified on day 28 were higher than on day 1 in both sexes, but especially in females. In any case, because no quantifiable concentrations were detected in the day 28 pre-dose and 24-h post-dose samples, the increased drug exposure in females at day 28 is more likely related to a defect in drug elimination than to accumulation of SET-M33.

Systemic exposure of dogs to SET-M33 generally appeared to have nonlinear (dose-dependent) kinetics in the dose range 0.5 to 4.0 mg/kg/day on days 1 and 28 of the 4-week intravenous (infusion) study. Increasing the dose of SET-M33 above 0.5 mg/kg/day is likely to result in higher systemic exposure than would be predicted in the case of a linear relationship. However, the C_max_ of SET-M33 in male dogs appeared to have linear (dose-independent) kinetics. In addition, the data also provided evidence that there were no differences in systemic exposure of male and female dogs to SET-M33 at the 0.5 and 1.5 mg/kg/day dose levels, but that at the highest dose (4.0 mg/kg/day), the systemic exposure of female dogs to SET-M33 was higher than that of males. Systemic exposure was higher after repeated intravenous administration (infusion) of SET-M33 than after a single dose and suggests that SET-M33 has time-dependent kinetics.

Daily intravenous infusion of the peptide at ≥ 0.5 mg/kg/day in beagle dogs did not produce any observable effect on heart function, as demonstrated by ECG parameters (heart rate, PR, QRS, QT and QTc intervals) in males and females (data not reported here).

In both animal species, daily administration of SET-M33 at the highest doses used in our tests caused renal effects, such as tubule degeneration/regeneration, elevated blood concentrations of urea and creatinine, high glucose (only in rats), all suggesting a functional deficit and identifying the kidneys as a possible target for toxic effects. This confirms the bio-distribution and excretion data obtained previously with radio-iodinate SET-M33^24^, which showed an evident uptake of the peptide by kidneys and bladder after intravenous administration of the peptide to mice. The bioanalytical evaluation of blood parameters and the histological analysis of the present study did not suggest any possible toxic effects on other organs in rats and dogs.

Dogs remain the main non-rodent species used in preclinical drug development^40^. Determination of the NOAEL in these animals is an important part of non-clinical risk assessment^40,41^. The results obtained with SET-M33 in the 4-week toxicity study with 4-week recovery period in dogs, especially regarding the adverse findings of renal tubule degeneration/regeneration and moderate to marked vascular thrombi at infusion sites at doses ≥ 1.5 mg/kg/day, indicate that the NOAEL is below this latter dosage, presumably around 0.5 mg/kg/day. This dose will be a useful starting point for an eventual phase I clinical trial.

All the studies reported in this article were designed to comply with accepted pharmacological principles and the requirements of Europe, Japan and the USA^30,31,42–49^.

## Methods

### SET-M33 peptide

Peptide with a purity of 97.9% as declared by the producer (Polypeptide, Strasbourg) was used for all tests. The formulations were prepared under sterile conditions by dissolving the powder in the vehicle (0.9% sodium chloride) and then sterile filtering with a 0.22 micron PVDF filtration unit. Dose formulations of SET-M33 and 0.9% sodium chloride for injection were analysed to confirm that the prepared dose formulations were homogeneous and that the administered SET-M33 concentrations were appropriate under the study conditions. The analytical method validated at the Testing Facility involved dilution of SET-M33 dose formulation samples in 100% water followed by quantification using high performance liquid chromatography with ultraviolet detection (HPLC–UV).

### Animals

#### Rats

Sprague Dawley rats were used for the toxicity studies. The animals were supplied by Envigo RMS S.L. All animals were 6–8 weeks old at the start of treatment and were allocated randomly to the treatment groups. The peptide was administered as an intravenous bolus in approximately 50 s via the lateral tail vein using a graduated syringe and a 24G (0.55 × 25 mm) needle. The administration volume was 5 mL/kg body weight.

#### Dogs

Naïve beagle dogs were used for the toxicity studies. The animals were supplied by Marshall US. They were 9–10 months old at the start of treatment with a body-weight range of 9.1–10 kg for the DRF study. They were 6–7 months old at the start of treatment with a body-weight range of 6.6–8.6 kg for males and 5.1–6.5 kg for females for the 4-week toxicity study. The method of administration was an intravenous short infusion with an infusion pump, alternately either into the cephalic or saphenous veins using sterile disposable cannulas and syringes. The volume of the dose of SET-M33 and control in two studies was 2, 5 and 5 mL/Kg body weight, respectively.

All experimental protocols were approved by licensing committees from the institutions where the experiments were carried out. All animal experiments were performed in collaboration with the following CROs: Covance CRS LLC (now Labcorp Drug Development), Huntingdon, Cambridgeshire UK and Somerset, New Jersey USA; AnaPath Research S.A.U., Castellar de Vallès, Barcelona, Spain.

All experimental procedures were carried out in accordance with the following guidelines and regulations: the United Kingdom Animals (Scientific Procedures) Act 1986 Amendment Regulations 2012; USA Animal Welfare Act Regulations: 9 CFR Parts 1 and 2 Final Rules, Federal Register, Volume 54, N. 168, August 31, 1989, pp. 36,112–36,163 effective October 30, 1989 and 9 CFR Part 3 Animal Welfare Standards; Final Rule, Federal Register, Volume 56, N. 32, February 15, 1991, pp. 6426–6505 effective March 18, 1991; Decret (Decree) 214/1997 of 30 July, Ministry of Agriculture, Livestock and Fishing of the Autonomous Government of Catalonia; Directive 2010/63/EU of the European Parliament and of the Council of 22 September 2010 on the protection of animals used for scientific purposes; Law 5/1995 of 21 June on the protection of animals used for experimentation and other scientific purposes (DOGC 2073, 10.7.1995), Autonomous Government of Catalonia; Law 6/2013 of 11 June, amending Law 32/2007 of 7 November on the care of animals during their exploitation, transport, experimentation and sacrifice, Spain; Real Decreto (Royal Decree) 53/2013 of 1 February 2013, Spain.

All methods are reported in accordance with ARRIVE guidelines (Animal Research: Reporting of In Vivo Experiments)^50^.

The number of animals used was the minimum consistent with scientific integrity and regulatory acceptability, considering the welfare of individual animals in relation to the number and extent of procedures to be carried out on each animal.

### Dose range finding (DRF) in rats

A viability/mortality check was recorded at least twice daily. Detailed observation of clinical signs was made daily during dosing.

#### Haematology

Blood samples were drawn from the retro-orbital plexus of all animals under light isoflurane anaesthesia. Blood was collected into tubes containing EDTA-K_3_ as anticoagulant. The following parameters were determined using an ADVIA 120 haematology analyser (Siemens Healthcare): Red blood cell count (RBC), haemoglobin (Hb), haematocrit (Hct), mean corpuscular volume (MCV), mean corpuscular haemoglobin (MCH), mean corpuscular haemoglobin concentration (MCHC), reticulocyte count (absolute and relative) (Retic), platelet count (Plt), total leukocyte count (WBC), neutrophils (N), lymphocytes (L), monocytes (M), eosinophils (E), basophils (B), large unstained cells (LUC).

#### Clinical biochemistry

Blood samples were collected into lithium heparin tubes. The plasma was analysed for the following parameters with a Cobas 6000 analyzer (Roche): glucose (Gluc), urea (Urea), creatinine (Creat), bilirubin, total (Bili), cholesterol, total (Chol), triglycerides (Trig), aspartate aminotransferase (AST), alanine aminotransferase (ALT), creatine kinase (CK), gamma-glutamyl-transferase (gGT), calcium (Ca), inorganic phosphorus (Phos), sodium (Na), potassium (K), chloride (Cl), total protein (total Prot), protein electrophoretogram, albumin (Alb), globulin (calculated from the total protein and Alb%) (Glob), album/globulin ratio (A/G ratio).

#### Urinalysis

Urine was collected in specimen vials using a metabolism cage. The following parameters were determined using a Cobas u 411 semi-automated test strip analyser (Roche): specific gravity (SG1), volume (Vol), colour (Col), appearance (App), pH, nitrite (Nite), protein (Prot), glucose (U-Gluc), ketones (Keto), urobilinogen (Urob), bilirubin (Bili), erythrocytes (U-RBC), leukocytes (U-WBC).

#### Necroscopy

Necroscopy was performed after the end of treatment in Phases I and II. Animals were sacrificed by intraperitoneal injection of sodium pentobarbital and immediately exsanguinated. Gross necropsy examination of the cranial, thoracic and abdominal cavities, major organs and injection site was performed on Phase I animals. A full necropsy was performed on Phase II animals, including examination of the external surface of the body, all orifices, cranial, thoracic and abdominal cavity organs in situ and after evisceration.

#### Bioanalytic and toxicokinetic study

Blood samples were collected from the retro-orbital plexus of animals under light isoflurane anaesthesia. Blood samples were taken from three males and three females at each extraction time. Sampling times were as follows: Day 1: 5, 15, 30 and 60 min after administration. Day 7: pre-dose and 5, 15, 30, 60 min and 24 h after administration. Control animals were bled only once on days 1 and 7 of treatment at 30 min after treatment. Each blood sample was collected into a polypropylene test tube containing lithium heparin as anticoagulant and kept in an ice bath until centrifuging (1600 g for 10 min at 2–8 °C). The plasma from each sample was transferred to a fresh polypropylene test tube, immediately frozen in dry ice and stored at − 20 °C ± 5.

### Four-week toxicity study with 2-week recovery period in rats

Viability/mortality was monitored twice daily throughout the study. Detailed clinical signs were evaluated once at pre-test, once/twice daily during the treatment period and on days 1, 8, 10 to 14 of the recovery period. Body weight was monitored once at pre-test, twice weekly during treatment and recovery periods, at termination of treatment and before sacrifice (scheduled animals fasted).

#### Haematology

Blood samples were extracted from the retro-orbital plexus under light isoflurane anaesthesia. The sample were collected into tubes containing EDTA-K_3_ anticoagulant. The following parameters were determined with an Advia 120 haematology analyser (Siemens Healthcare): red blood cell count (RBC), haemoglobin (Hb), haematocrit (Hct), mean corpuscle volume (MCV), red cell volume distribution width (RDW), mean corpuscular haemoglobin (MCH), mean corpuscular haemoglobin concentration (MCHC), haemoglobin concentration distribution width (HDW), reticulocyte count (Retic), platelet (thrombocyte) count (Plt), leukocyte count, total (WBC), neutrophils (N), lymphocytes (L), monocytes (M), eosinophils (E) basophils (B), lrge unstained cells (LUC).

#### Coagulation

Blood samples were collected into 3.2% sodium citrate tubes to obtain the plasma. The following parameters were determined using a STA COMPACT Automatic Coagulometer: prothrombin time (SPT), activated partial thromboplastin time (SAPT).

#### Clinical biochemistry

Blood samples were collected into lithium heparin tubes. The following parameters were determined using a Cobas 6000 Analyzer (Roche): glucose (Gluc), urea (Urea), creatinine (Creat), bilirubin, total (Bili), cholesterol, total (Chol), triglycerides (Trig), aspartate aminotransferase (AST), alanine aminotransferase (ALT), alkaline phosphatase (ALP), creatine kinase (CK), gamma-glutamyl-transferase (gGT), calcium (Ca), inorganic phosphorus (Phos), sodium (Na), potassium (K), chloride (Cl), albumin (Alb), globulin (calculated from total protein and Alb%) (Glob), total protein (Total Prot), album/globulin ratio (A/G ratio).

#### Urinalysis

Urine was collected overnight into specimen vials (animals were placed in metabolism cages at the end of the working day preceding the day of urine collection). The following parameters were determined using a Cobas u 411 semi-automated test strip analyser (Roche): specific gravity (SG1), protein (Prot), ketones (Keto), volume (Vol), creatinine (U-Creat), bilirubin (Bili), colour (Col), glucose (U-Gluc), erythrocytes (U-RBC), appearance (App), nitrite (Nite), pH, leukocytes (U-WBC).

#### Necroscopy

All animals underwent necropsy. Descriptions of all macroscopic abnormalities were recorded. Samples of tissues and organs were collected from all animals, weighed and fixed in neutral phosphate-buffered 4% formaldehyde solution (10% formalin). All organ and tissue samples to be examined were processed, embedded, cut and stained with haematoxylin and eosin.

### Neurological toxicity in rats

Evaluation of SET-M33 neurological toxicity was based on the method described by Irwin^32,33^. On the day prior to dosing, detailed subjective observation of all animals was made to assess the neurobehavioral and physiological state of untreated rats. After subjective observation, rectal temperature was measured and spontaneous locomotor activity was assessed. On the day of dosing, the animals were treated and then returned to their home cages. Detailed subjective observation of the rats was then repeated 5, 30, 90 and 240 min after dosing. A further observation was made 24 h after dosing. During these observations, the following parameters were systematically evaluated for each animal using a standard procedure: lethality, restlessness, apathy, writhing, fighting, stereotyped behaviour, tremor, twitches, convulsions, exophthalmos, abnormal respiration, alertness, startle response, loss of righting reflex, abnormal body carriage, abnormal gait, Straub tail, piloerection, pupil diameter, light-pupil response, touch response, fearfulness, pinna reflex, corneal reflex, catalepsy, passivity, aggressiveness, body tone, grip strength, cutaneous blood flow, cyanosis, ptosis, lacrimation, salivation, pain response, motility impairment, grooming, diarrhoea, vocalization, increased urination. Normal attributes of animals (e.g. alertness, body tone etc.) were subjectively scored as 4; enhancement or depression of these attributes by SET-M33 was scored with higher or lower integers, respectively. Attributes normally absent in animals (e.g. abnormal gait, abnormal respiration, tremors etc.) were subjectively scored from 0 (normal) to 8. At the end of each observation period, the rectal temperature of each animal was measured. Finally, spontaneous locomotor activity was analysed. Each animal was placed in a suitable arena and locomotor activity measured in terms of number of squares crossed in a 2-min period. Animals were inspected daily from day 3 to day 7 for appearance of any delayed effects. After the day 7 inspection, the animals were killed humanely by a rising concentration of carbon dioxide. Death was confirmed by dislocation of the neck.

### Evaluation of respiratory function in rats

Whole body, bias flow plethysmography equipment was used^51^. Respiratory parameters (respiratory rate, tidal volume and minute volume) were derived from the changes in pressure associated with the warming and humidification of the air breathed in by the animal. This was monitored by specific probes located in the plethysmograph chambers. Bias flow (room air) was set at approximately 2.5 L/min. On a day prior to the first day of dosing, all animals were habituated to the plethysmographs for approximately 2 h. The study was run over 4 days and 10 animals (two from each group) were examined each day. On the day of dosing, the animals were placed in the plethysmographs for pre-dose recording (session 1) of respiratory parameters for 60 min. Groups of 8 rats were then removed from the chambers and dosed by intravenous bolus injection with vehicle, SET-M33 or positive control. Immediately after dosing, the rats were placed in the whole body plethysmographs, where they could move about freely, to continuously record respiratory parameters for 4 h post-dose (undisturbed recording). Immediately after the last post-dose recording, the animals were killed humanely by a rising concentration of carbon dioxide. Death was confirmed by dislocation of the neck. Respiratory parameters were reported at the following time points: 0, 30, 60, 90, 120, 150, 180, 210 and 240 min. Time 0 coincided with the mean value of the last 20 min of data recorded in the 60-min pre-dose period. All other time points were the mean of 10-min recordings (an average of every 10 breaths) around each time point. Each post-dose time point was analysed separately by analysis of covariance. Factors in the model were group and day of data collection, with pre-dose values as covariate.

### Dose range finding (DRF) in dogs

Viability/mortality were recorded at least twice daily. On treatment days, all animals were observed for signs of toxic or pharmacological effects prior to, during and immediately after administration and 1–2 h post-dose. Injection sites were examined daily during dosing. For clinical investigation, blood obtained via jugular venepuncture from anaesthetized dogs was used to analyse blood, coagulation and biochemical parameters.

#### Haematology

A blood sample was collected into tubes containing EDTA-K_3_ as anticoagulant and analysed for the following parameters using Advia 120 haematology analyser (Siemens Healthcare): red blood cell count (RBC), haemoglobin (Hb), haematocrit (Hct), mean corpuscular volume (MCV), mean corpuscular haemoglobin (MCH), mean corpuscular haemoglobin concentration (MCHC), platelet count (Plt), total leukocyte count (WBC), reticulocyte count (Retic), neutrophils (N), lymphocytes (L), monocytes (M), eosinophils (E) basophils (B), large unstained cells (LUC).

#### Clinical biochemistry

A blood sample was collected in lithium heparin tubes. The following parameters were determined using Cobas 6000 analyzer (Roche): albumine (Alb), glucose (Gluc), urea (Urea), creatinine (Creat), bilirubin (Bili), cholesterol (Chol), aspartate aminotransferase (AST), alanine aminotransferase (ALT), sodium (Na), potassium (K), chloride (Cl), calcium (Ca), inorganic phosphorus (Phos), total protein (Total Prot), protein electrophoretogram.

#### Urinalysis

Urine obtained via a 16-h overnight collection period was analysed. Urine was collected into ice-chilled containers overnight from pans placed beneath each animal’s cage. Urine samples were analysed for the following parameters using Multistix reagent strips, interpreted using a Siemens Clinitek Advantus: specific gravity (SG1), colour (Col), pH, protein (Prot), glucose (U-Gluc), ketones (Keto), urobilinogen (Urob), bilirubin (Bili).

#### Necroscopy

All animals were sacrificed at the end of the treatment period by intravenous injection of sodium pentobarbital. Organs were collected and weighed.

#### Bioanalytic and toxicokinetic study

Blood samples were taken by direct venepuncture of the jugular vein on days 1 and 7 to determine plasma levels of SET-M33. Sampling times were as follows: Day 1: at 0, 5, 15, 30, 60 and 180 min. Day 7: at 0, 5, 15, 30, 60, 180 min and 24 h after administration. Control animals were only bled once on days 1 and 7 of treatment, 1 h after administration. Blood samples were collected into lithium heparin test tubes and kept in an ice bath until centrifuging (1600 g for 10 min at 2–8 °C). The plasma obtained from each sample was transferred to a fresh polypropylenes test tubes, immediately frozen in dry ice and stored at − 20 °C ± 5.

### Four-week toxicty study with 4-week recovery period in dogs

Animals were observed daily for mortality and signs of severe toxic or pharmacological effects. On treatment days, all animals were observed for signs of toxic or pharmacological effects prior to, during and after administration and 1–2 h post-dose*.* Blood obtained via jugular venepuncture from anaesthetized dogs was used to analyse blood, coagulation and clinical chemistry parameters for 5 animals/sex/group pre-test and at termination of dosing and from 2 animals/sex/group at the end of recovery.

#### Haematology

Blood samples were collected into tubes containing K_3_EDTA anticoagulant and analysed for the following using ADVIA 120 Haematology Analyser (Siemens): haemoglobin (Hb), haematocrit (Hct), red blood cell count (RBC), platelet count (Plt), mean corpuscular volume (MCV), mean corpuscular haemoglobin (MCH), mean corpuscular haemoglobin concentration (MCHC), red cell distribution width (RDW), total white blood cell count (WBC), reticulocyte count (Retic), neutrophils (N), lymphocytes (L), eosinophils (E), basophils (B), monocytes (M), large unstained cells (LUC).

#### Coagulation

Blood samples were collected into tubes containing sodium citrate anticoagulant and analysed for the following using a Diagnostica Stago Products STA Compact MAX mechanical clot detection system: prothrombin time (SPT), activated partial thromboplastin time (APTT), fibrinogen (FIB).

#### Clinical biochemistry

Blood samples were collected into tubes with no anticoagulant, allowed to clot, centrifuged to obtain serum and analysed for the following using ADVIA 1800 Chemistry Analyzer (Siemens): aspartate aminotransferase (AST), alanine aminotransferase (ALT), alkaline phosphatase (ALKP), blood urea nitrogen (BUN), creatinine (Creat), glucose (Glu), cholesterol (Chol), triglycerides (Trig), total protein (Tot Prot), albumin (Alb), total bilirubin (Bili), sodium (Na), potassium (K), chloride (Cl), calcium (Ca), inorganic phosphorus (Phos), gamma-glutamyl transferase (gGT).

#### Urinalysis

Urine obtained via a 16-h overnight collection period was analysed for all animals/sex/group pre-test, at study termination, and at the end of recovery. Urine was collected into ice-chilled containers overnight from pans placed beneath each animal’s cage. Urine samples were analysed for the following parameters using Multistix reagent strips, interpreted using a Siemens Clinitek Advantus: pH, protein (Prot), glucose (U-Gluc), ketones (Keto), bilirubin (Bili), appearance (App), specific gravity (SG1), volume (Vol).

#### Necroscopy

Complete macroscopic examination was performed on all animals, including examination of the external surface and all orifices; the external surfaces of the brain and spinal cord; the organs and tissues of the cranial, thoracic, abdominal and pelvic cavities and neck; and the rest of the carcass for macroscopic morphological abnormalities. Necropsy was performed on up to 3 animals/sex/group after treatment for 4 weeks and on 2 animals/sex/group after a 4-week treatment-free recovery period. Organs and tissues were weighed, preserved and examined microscopically. Prior to weighing, organs were carefully dissected and properly trimmed to remove adipose and other contiguous tissue in a uniform manner. Organs were weighed as soon as possible after dissection in order to avoid drying. Paired organs were weighed together. Eyes, optic nerve and testes were initially placed in Modified Davidson’s solution and then kept in 10% neutral buffered formalin (NBF). Lungs and urinary bladder were infused with 10% NBF prior to immersion in a larger volume of the same fixative. All other tissues were preserved in 10% NBF.

### Bioanalytics and toxicokinetics in rats and dogs

#### Rats

Blood samples were drawn from the retro-orbital sinus under light isoflurane anaesthesia for determination of plasma SET-M33 levels. The samples were taken on days 1 and 28 of treatment at the following times: control group: pre-dose and 30 min; 5, 9 and 15 mg/kg/day-group: pre-dose, 5, 15, 30, 60 min and 24 h after administration. Blood samples were collected into polypropylene test tubes containing lithium heparin anticoagulant and kept at room temperature for no longer than 60 min until centrifuging (1600 g for 10 min at 2–8 °C). The plasma obtained from each sample was transferred to a fresh polypropylene test tubes, immediately frozen in dry ice and stored at − 20 °C ± 5. Plasma concentrations of SET-M33 were measured by a previously validated LC–MS/MS method. Toxicokinetic parameters were determined for mean plasma concentrations at each dose level and time point, according to the validated method and with WinNonlin software, version 6.3, in the Phoenix Suite version 1.3 (Pharsight Corporation, Mountain View, CA, USA).

#### Dogs

On days 1 and 28, blood samples were obtained for toxicokinetic determinations from all animals at the following times: Day 1 at the end of infusion and 5, 10, 15, 30, 60 and 180 min later; 6 ± 15 min and 24 h ± 30 min post-dose; Day 28 at the end of infusion and 5, 10, 15, 30, 60 and 180 min later; 6 ± 15 min and 24 h ± 30 min post-dose. Blood samples were collected into lithium heparin test tubes and kept in an ice bath until centrifuging (1600 g for 10 min at 2–8ºC). The plasma obtained from each sample was transferred to a fresh polypropylenes test tubes, immediately frozen in dry ice and stored at − 20ºC ± 5. Bioanalytical samples were analysed by a validated liquid chromatographic mass spectrometric assay. Pharmacokinetic parameters were calculated using the computer program Phoenix WinNonlin version 6.3 (Certara USA, Inc).

## Supplementary Information

Supplementary Information 1.

Supplementary Information 2.

## Data Availability

The datasets used and/or analysed during the current study are available from the corresponding author on reasonable request.
