# Supplementary material for: Safety evaluations of a synthetic antimicrobial peptide administered intravenously in rats and dogs
Source: Sci Rep. 2022 Nov 11;12:19294. doi: 10.1038/s41598-022-23841-2 (PMC9652379; doi:10.1038/s41598-022-23841-2)
Supplement: Supplementary file 1 — Supplementary Information 1. [file 41598_2022_23841_MOESM1_ESM.pdf]

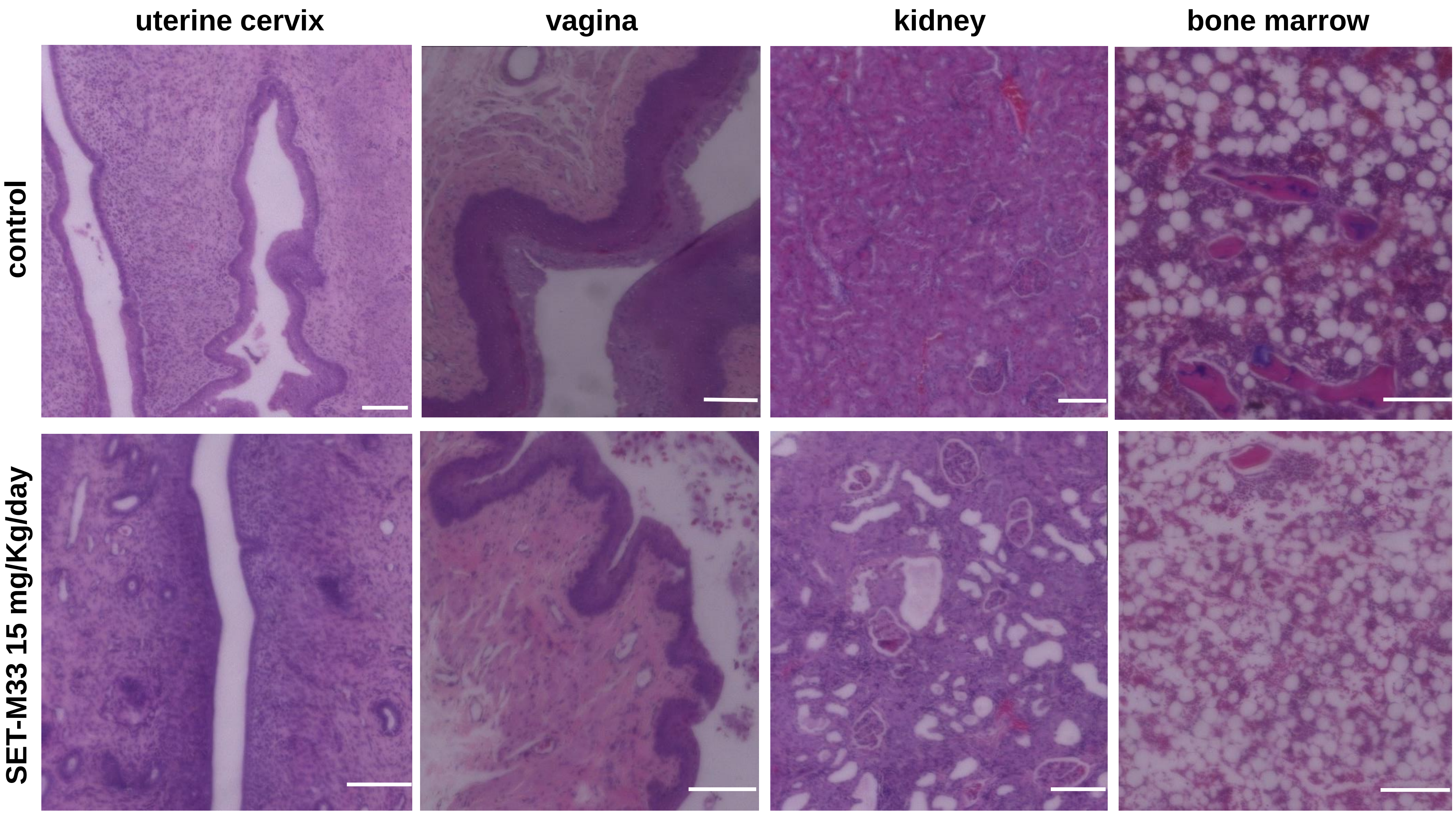

Histological sections of tissues collected after 2-week recovery period of **rats** treated with vehicle (panels above) and SET-M33 at 15 mg/Kg/day for 28 days (panels below). In the animals treated with SET-M33 the following findings resulted evident: atrophy of the epithelial surface in uterine cervix and vagina; parenchymal degenerations in the kidney; increase of adipocytes in the femur bone marrow. Scale bars (white lines) correspond to 100 µm.

Samples were examined by confocal laser microscope (Leica TCS SP8). All images were processed using ImageJ software (NIH)
