# Supplementary material for: Safety evaluations of a synthetic antimicrobial peptide administered intravenously in rats and dogs
Source: Sci Rep. 2022 Nov 11;12:19294. doi: 10.1038/s41598-022-23841-2 (PMC9652379; doi:10.1038/s41598-022-23841-2)
Supplement: Supplementary file 2 — Supplementary Information 2. [file 41598_2022_23841_MOESM2_ESM.docx]

Notes about irreversible abnormalities in rats

**Kidneys**

In all treated animals (15 mg/Kg/day) severe nephropathy was present. Therefore, changes were considered not to have resolved after the two-week recovery period.

**Bone marrow**

Both in the femur and sternum of 3 out of 5 males and 2 out of 5 females treated at 15 mg/kg/day, a

minimal to slight increase in adipocytes was observed in the bone marrow, compared with concurrent controls. This change was not considered to have reversed, but rather increased in incidence compared with the end of treatment.

**Uterine Cervix and Vagina**

1 out of 5 animal treated at 15 mg/kg/day showed cervical and vaginal epithelial atrophy compared to controls. Therefore, vaginal changes were considered not to have resolved.
